# Assembly and proteolytic processing of mycobacterial ClpP1 and ClpP2

**DOI:** 10.1186/1471-2091-12-61

**Published:** 2011-12-01

**Authors:** Nadia Benaroudj, Bertrand Raynal, Marika Miot, Miguel Ortiz-Lombardia

**Affiliations:** 1 Institut Pasteur, Unité de Biologie des Spirochètes, Institut Pasteur, F-75015 Paris France; 2 Institut Pasteur, Plate-Forme de Biophysique des Macromolécules et de leurs Interactions, Institut Pasteur, F-75015 Paris France; 3 Laboratory of Molecular Biology, National Cancer Institute, National Institutes of Health, Bethesda, MD 20892, USA; 4 Architecture et Fonction des Macromolécules Biologiques (UMR6098), CNRS, Universités d'Aix-Marseille I & II, 163 Avenue de Luminy, 13288 Marseille cedex 9, France

## Abstract

**Background:**

Caseinolytic proteases (ClpPs) are barrel-shaped self-compartmentalized peptidases involved in eliminating damaged or short-lived regulatory proteins. The *Mycobacterium tuberculosis *(MTB) genome contains two genes coding for putative ClpPs, ClpP1 and ClpP2 respectively, that are likely to play a role in the virulence of the bacterium.

**Results:**

We report the first biochemical characterization of ClpP1 and ClpP2 peptidases from MTB. Both proteins were produced and purified in *Escherichia coli*. Use of fluorogenic model peptides of diverse specificities failed to show peptidase activity with recombinant mycobacterial ClpP1 or ClpP2. However, we found that ClpP1 had a proteolytic activity responsible for its own cleavage after the Arg8 residue and cleavage of ClpP2 after the Ala12 residue. In addition, we showed that the absence of any peptidase activity toward model peptides was not due to an obstruction of the entry pore by the N-terminal flexible extremity of the proteins, nor to an absolute requirement for the ClpX or ClpC ATPase complex. Finally, we also found that removing the putative propeptides of ClpP1 and ClpP2 did not result in cleavage of model peptides.

We have also shown that recombinant ClpP1 and ClpP2 do not assemble in the conventional functional tetradecameric form but in lower order oligomeric species ranging from monomers to heptamers. The concomitant presence of both ClpP1 and ClpP2 did not result in tetradecameric assembly. Deleting the amino-terminal extremity of ClpP1 and ClpP2 (the putative propeptide or entry gate) promoted the assembly in higher order oligomeric species, suggesting that the flexible N-terminal extremity of mycobacterial ClpPs participated in the destabilization of interaction between heptamers.

**Conclusion:**

Despite the conservation of a Ser protease catalytic triad in their primary sequences, mycobacterial ClpP1 and ClpP2 do not have conventional peptidase activity toward peptide models and display an unusual mechanism of self-assembly. Therefore, the mechanism underlying their peptidase and proteolytic activities might differ from that of other ClpP proteolytic complexes.

## Background

In all organisms, ATP-dependent proteases play an essential role by removing short-lived regulatory proteins whose rapid elimination is critical for cell metabolism and growth [1]. They also allow riddance of misfolded and damaged proteins that accumulate in a variety of circumstances, notably during environmental stress [2]. Simultaneous orchestrated action of key regulatory proteins as well as protein quality control mechanism assure successful survival and virulence of bacteria.

The Clp (caseinolytic protease) proteolytic system is one of the major ATP-dependent proteolytic complexes in bacteria. It consists of a barrel-shaped tetradecameric ClpP peptidase organized in two-stacked heptameric rings. The active sites for peptide bond cleavage are sequestered inside a proteolytic chamber accessible only through narrow axial pores and whose entry is restricted to unfolded polypeptides [3]. In *E.coli*, where the biochemical properties of ClpP have been extensively studied, the peptidase can on its own efficiently degrade small peptides of up to six amino acid residues [4,5]. Degradation of longer peptides and proteins required the interaction of ClpP with an ATPase complex, namely ClpA or ClpX in most Gram negative bacteria and ClpX, ClpC, and possibly ClpE and ClpL in Gram positive bacteria [6]. This ATPase complex, a ring-shaped hexamer that aligns coaxially with the ClpP peptidase tetradecamer, allows recognition of polypeptide substrates and, through ATP hydrolysis, provides the energy required for protein unfolding and translocation inside the proteolytic chamber where the polypeptide chain is degraded to small peptides.

*Mycobacterium tuberculosis *(MTB), the etiologic agent of tuberculosis in humans, is one of the deadliest pathogens on earth, killing nearly 2 million people each year [7].

MTB has unique biological properties that enable it to persist in phagosomes for decades in a poorly understood latent form and from where it can be reactivated and cause active tuberculosis [8]. The phagosome is a hostile environment which is acidic, nutrient and oxygen poor, and oxidative and nitrosative due to the production of reactive oxygen and nitrogen species by the host [9]. Consistent with a powerful adaptive capacity, microarray studies have shown that MTB survival in macrophages is accompanied by changes in expression of about 600 genes, many of them involved in starvation, nitrosative and oxidative stress responses [10].

One of the factors potentially involved in the pathogenesis of MTB is the ClpP proteolytic complex, and growing evidences have involved this protease in the virulence of numerous other pathogen bacteria including *Salmonella typhimurium *[11], *Listeria monocytogenes *[12], *Streptococcus pneumoniae *[13], *Staphylococcus aureus *[14], and *Helicobacter pylori *[15].

The complete genome sequencing of the best-characterized *M. tuberculosis *strain (H37Rv) has revealed the presence of two paralog genes encoding putative ClpP peptidase, *clpP1 *(Rv2461c) and *clpP2 *(Rv2460c) (Pasteur Institute TubercuList, http://genolist.pasteur.fr/TubercuList), probably organized as an operon [16].

Presence of multiple clpP genes is common to actinomyces, whereas most other bacteria contain only one *clpP *gene. *clpP2 *has been predicted as essential for growth of MTB by transposon mutagenesis [17]. Both genes have their expression up-regulated during reaeration of MTB cultures [16] and are important for MTB to replicate in macrophages [18]. Despite their potential importance in survival and virulence of MTB, to our knowledge, functional studies of these peptidases have not yet been reported.

In this study, in order to gain insight into the mechanism of protein degradation by ClpPs from MTB, we have produced and purified recombinant ClpP1 and ClpP2 and tested their peptidase activities. No conventional chymotryptic activity could be detected toward the model peptide Suc-LY-Amc (N-Succinyl-Leu-Tyr-7 amido 4 methylcoumarin) under conditions that normally favor ClpP activity. However, ClpP1 was shown to have a proteolytic activity responsible for its own cleavage after the Arg8 residue and cleavage of ClpP2 after the Ala12 residue. We have investigated whether the cleavage of model peptides by ClpP1 and ClpP2 would require their proteolytic processing, deletion of the amino termini that could prevent peptide entry, or the presence of the ATPases ClpC and ClpX. We have also tested how the truncations in the amino-termini of the proteins would change their oligomeric assembly and found that they led to a higher order of assembly.

## Results

### Recombinant ClpP1 and ClpP2 do not cleave the Suc-LY-Amc peptide but can inhibit *E. coli *ClpP activity

The Rv2461c and Rv2460c genes encode proteins of respectively 200 and 214 amino acids annotated as ClpP1 and ClpP2. Alignment of the primary sequence of those proteins with that of *E. coli *ClpP shows a conservation of the Ser, His, and Asp catalytic triad residues (see additional file 1), indicating that they both have a putative Ser peptidase activity.

**Figure 1:**
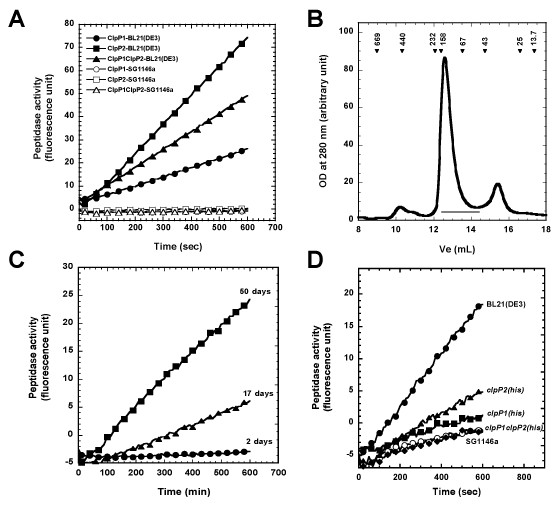
**ClpP1 and ClpP2 interact with *E. coli *ClpP and inhibit its peptidase activity**. (A) Hydrolysis of the Suc-LY-Amc peptide was carried out as described in the Methods section with 5 μg (black triangles) or 10 μg (all other samples) of the indicated purified proteins produced in BL21(DE3) (closed symbols) or in SG1146a cells (open symbols). (B) Size-exclusion chromatography of recombinant ClpP2 purified from BL21(DE3) when expressed as the *clpP1-clpP2(his)_6 _*operon. 500 μg of purified ClpP2(His)_6 _was loaded on a Superdex 200 10/30 column as described in the Methods section. Arrowheads indicate the elution of molecular mass standards with their molecular mass in kDa. The horizontal bar indicates the fractions that were collected and pooled for measurement of peptidase activity in Figure 1C. (C) Hydrolysis of the Suc-LY-Amc peptide by 10 μg of the recombinant ClpP2(His)_6 _purified by SEC as described in (B) after 2 (black circles), 17 (black triangles), and 50 (black squares) days of storage at 4°C. (D) Hydrolysis of the Suc-LY-Amc peptide by 10 μg of total extracts of SG1146a cells overexpressing the pET26b plasmid (open circles) or BL21(DE3) cells overexpressing the pET26b plasmid (black circles), the pET26b plasmid carrying the *clpP1(his)_6 _*(black squares) or the *clpP2(his)*_6 _(black triangles) open reading frames, or the *clpP1-clpP2(his)_6 _*operon (black diamonds).

In order to study the mycobacterial ClpP peptidase activities, *clpP1(his)_6_*, *clpP2(his)_6 _*and a *clpP1-ClpP2(his)_6 _*operon were expressed under the control of T7 promoter in *E. coli *BL21(DE3) cells. When the corresponding proteins were purified by a single Ni^2+ ^affinity chromatography and left for several weeks at 4°C, all the preparations exhibited a peptidase activity toward the Suc-LY-Amc fluorogenic peptides (Figure 1A). However, replacing the active site serine residue of ClpP1 (Ser 98) and ClpP2 (Ser 110) by alanine residues did not abolish the observed peptidase activity (data not shown). When ClpP1 and ClpP2 were produced in *E. coli *cells lacking endogenous ClpP (SG1146a strain), no peptidase activity toward the Suc-LY-AMC peptide was detected (Figure 1A). Also, when an extract was prepared from empty vector-expressing BL21(DE3) cells and subjected to a Ni^2+ ^affinity chromatography, and incubated with the Suc-LY-Amc peptide, no peptidase activity was found (see additional file 2). Therefore, *E. coli *ClpP rather than MTB ClpP1 and ClpP2 was responsible for the observed peptidase activities and the co-purification of *E. coli *ClpP depends on the presence of ClpP1 or ClpP2.

It is noteworthy that not all protein preparations had the same specific peptidase activity toward Suc-LY-Amc nor it could be detected after the same duration of incubation at 4°C. The proteins purified when the *ClpP1-ClpP2(his)_6 _*operon was overexpressed showed the most rapidly detectable peptidase activity (note that, in the Figure 1A, only 5 μg of ClpP1/ClpP2(His)_6 _were used whereas 10 μg of the other preparations were used), and the ClpP1(His)_6 _preparation needed to be incubated a longer time at 4°C in order to detect *E. coli *ClpP peptidase activity.

In order to eliminate the contaminating *E. coli *ClpP peptidase activity, the proteins purified when the *ClpP1-ClpP2(his)_6 _*operon was overexpressed were subjected to a second chromatographic step. The elution fraction of the Ni^2+ ^column contained mainly ClpP2(His)_6 _and a small amount of ClpP1 (data not shown). When subjected to a size exclusion chromatography (SEC), this protein mixture eluted as a main peak with an elution volume of 12.6 mL, which corresponds to a species having an apparent molecular mass of about 104 kDa (Figure 1B). Active *E. coli *ClpP peptidase assembles in a tetradecameric complex of about 300 kDa. Therefore, this peak should not contain any active tetradecamer of *E. coli *ClpP. Fractions corresponding to the mycobacterial ClpP peak were pooled and stored at 4°C. As seen in Figure 1C, after two days no peptidase activity was detectable. After 17 days of storage at 4°C, a peptidase activity was measurable and this activity kept increasing after 50 days of storage. Comparable peptidase activity was observed if the protein mixture was not subjected to a SEC but no peptidase activity could be observed if the *ClpP1-ClpP2(his)_6 _*operon was overexpressed in the SG1146a strain and the proteins subjected to a SEC purification step (data not shown). This suggests that the major peak eluting from the SEC column at 12.6 mL contained, in addition to MTB ClpPs, inactive subunits of *E. coli *ClpP that could reassemble into an active complex upon storage at 4°C. This would imply that *E. coli *ClpP subunits could associate with MTB ClpP1 and ClpP2 to form complexes inactive toward the Suc-LY-Amc peptide. An interaction between *E. coli *ClpP and MTB ClpP1 or ClpP2 was demonstrated by co-producing ClpP1(His)_6 _or ClpP2(His)_6 _with untagged *E. coli *ClpP and showing that the binding of ClpP to a Ni^2+ ^resin depended on the MTB ClpP1 or ClpP2 (see additional file 3). To verify that ClpP1 or ClpP2, when interacting with *E. coli *ClpP, would inhibit ClpP peptidase activity, we tested whether expression of ClpP1 and ClpP2 could decrease cleavage of Suc-LY-Amc peptide by *E.coli *ClpP. As seen in Figure 1D, a peptidase activity toward Suc-LY-Amc peptide was detected in total extract of BL21(DE3) that could be attributed to *E. coli *ClpP since it dramatically decreased when *clpP *was inactivated (SG1146a strain). Expressing *clpP1(his)_6_*, *clpP2(his)_6_*, or the *clpP1-clpP2(his)_6 _*operon led to a decrease in *E. coli *ClpP peptidase activity, indicating that MTB ClpPs subunits may interact with *E. coli *ClpP subunits and inhibit its activity. Such kind of property has never been described for any other recombinant ClpPs produced in *E. coli *and might reflect a difference in the assembly pathway of protomers into tetradecamers. Our finding that the purified proteins released the active *E. coli *ClpP more rapidly when *clpP1 *and *clpP2 *are expressed from an operon than when MTB ClpPs are produced independently might indicate a stronger, perhaps physiological, interaction between ClpP1 and ClpP2. The copurification of ClpP1 with ClpP2 makes such a hypothesis very likely. Those findings demonstrated the need to produce MTB ClpP1 and ClpP2 in a bacterial strain that does not contain any endogenous *E. coli *ClpP.

Since we could not detect any chymotryptic peptidase activity by MTB ClpP1 and ClpP2 toward the Suc-LY-Amc peptide, we also tested cleavage of other peptides. ClpP1 and ClpP2, whether produced independently or from the *clpP1-clpP2(his)_6 _*operon, did not cleave the Bz-VGR-Amc (benzoyl-Val-Gly-Arg 7 amido 4 methylcoumarin) or Boc-LRR-Amc (terbutyloxycarbonyl-Leu-Arg-Arg 7 amido 4 methylcoumarin) peptides (data not shown), indicating that they do not exhibit trypsin-like activity toward those peptides in the conditions used in our assay.

### Recombinant MTB ClpP1 and ClpP2 do not assemble into tetradecamers in solution

**Figure 2:**
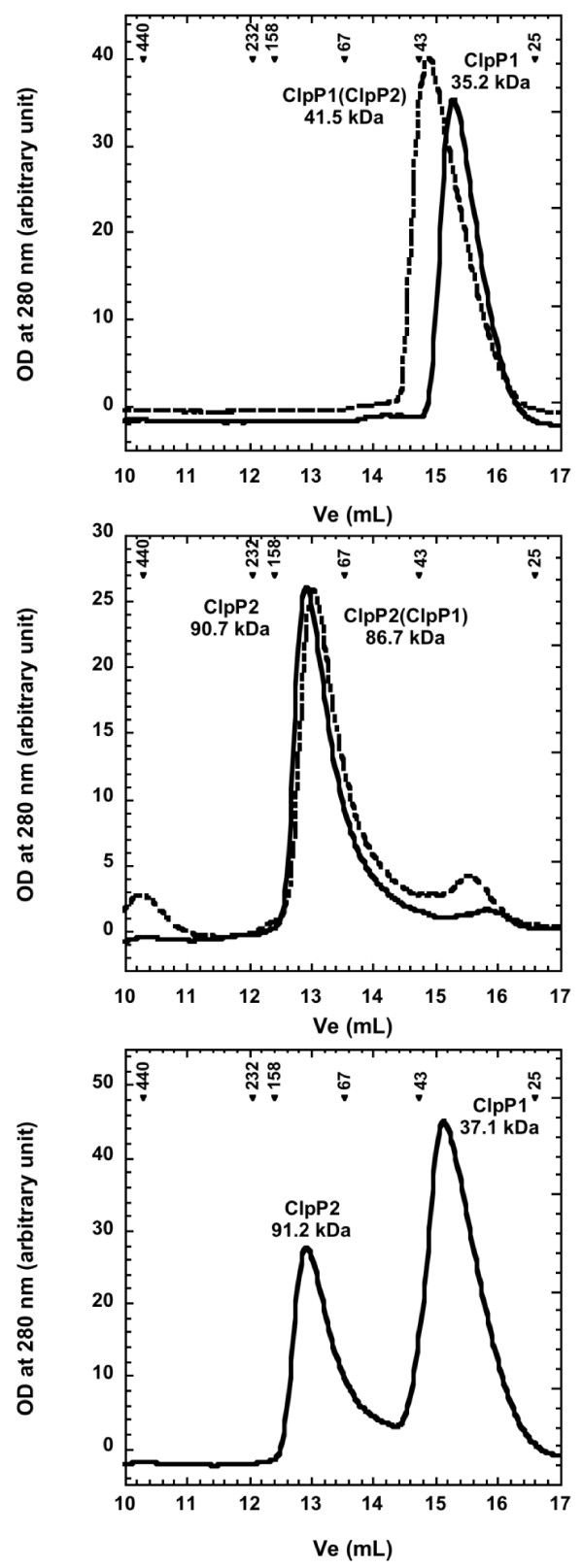
**Oligomeric assembly of recombinant ClpP1 and ClpP2**. 75 μg of recombinant ClpP1 (upper panel, solid line) or ClpP2 (middle panel, solid line) produced independently or in the presence of ClpP2 (upper panel, dashed line) and ClpP1 (middle panel, dashed line) respectively were loaded on a superdex 200 10/30 column as described in the Methods section. In the lower panel, 75 μg of ClpP1 were mixed with 75 μg of ClpP2 and incubated 2 h at room temperature before being loaded on the superdex 200 column. Arrowheads indicate the elution of molecular mass standards with their molecular mass in kDa and the deduced apparent molecular masses are shown under the corresponding protein names.

One of the hypotheses to explain the absence of any peptidase activity observed in our assay is the absence of a tetradecameric assembly. Therefore, the presence of a correct tetradecameric assembly was examined on purified ClpP1 and ClpP2. When subjected to SEC, purified ClpP1 eluted as a species having an apparent molecular mass of 35 kDa, compatible with monomers and dimers of ClpP1 (Figure 2, upper panel, solid trace). Purified ClpP2 eluted as a molecular complex having an apparent molecular mass of about 91 kDa (Figure 2, middle panel, solid trace). These results indicated that neither ClpP1 nor ClpP2 assembled in tetradecamers in these conditions.

The possibility that both ClpP1 and ClpP2 were needed to promote tetradecamer assembly was tested. As seen in Figure 2 (upper panel, dashed line), coproducing ClpP2 with ClpP1 did not dramatically change the elution volume of purified ClpP1 and similarly coproducing ClpP1 with ClpP2 did not influence the elution volume of ClpP2 (middle panel, dashed line). Furthermore, mixing purified ClpP1 and ClpP2 did not change the elution volumes of individual ClpP1 or ClpP2 (Figure 2, lower panel). Therefore, tetradecameric assembly was not mutually induced by the presence of either ClpP1 or ClpP2.

It is noteworthy that the presence of a His tag at the C-termini of ClpP1 and ClpP2 could not explain the absence of tetradecamer formation since producing and purifying untagged ClpP1 and ClpP2 led to the same oligomeric assembly as His tagged peptidases (data not shown).

### Peptidase activity and assembly of processed ClpP1 and ClpP2

We then explored different procedures to stimulate the peptidase activity and to promote correct tetradecameric assembly of ClpP1 and ClpP2.

In *E. coli*, ClpP matures as an active peptidase by the autoproteolytic removal of the first 14 amino acid residues [19,20]. In the X-ray structure of MTB ClpP1, the first 14 residues are not visible and it was suggested that the N-terminus of the protein was disordered and might prevent the entry of small peptides into the central proteolytic cavity, explaining the lack of peptidase activity [21]. We thus tested whether processed ClpP1 and ClpP2 would correctly assemble in tetradecamers and exhibit chymotryptic activity.

**Figure 3:**
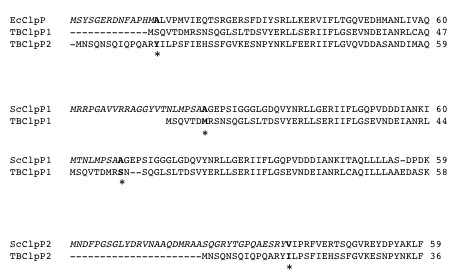
**Putative N-terminal processing sites for MTB ClpP1 and ClpP2**. The N-terminal sequences of MTB ClpP1 (H37Rv strain, gi: 41353667) and ClpP2 (H37Rv strain, gi: 2791500) were aligned with that of *E. coli *ClpP (gi: 89107307) and with those of *S*. coelicolor ClpP1 (gi: 10280519) and ClpP2 (10280518) respectively using ClustalW program http://www.ebi.ac.uk/Tools/msa/clustalw2. The propeptides of *E. coli *ClpP and *Streptomyces *ClpP1 and ClpP2 are written in italics and the first residues of mature ClpPs are indicated in bold. The putative first residues of MTB ClpP1 and ClpP2 are written in bold and indicated with an asterisk.

Processed *E. coli *ClpP has been shown to start with Ala15 [19]. Based on alignment with *E.coli *ClpP, ClpP1 is not expected to be processed (Figure 3). If aligned with the whole sequence of *Streptomyces *ClpP1 (a closer ortholog), it could be processed at Met7. However, it was shown that *Streptomyces *ClpP1 had another putative initiation codon according to the codon usage [22] and if aligned with the streptomyces ClpP1 sequence starting with this second putative initiation codon MTB ClpP1 would be processed at the Ser9 residue (Figure 3).

Based on alignement with *E.coli *ClpP, ClpP2 is predicted to be processed at Tyr14. In *Streptomyces*, mature ClpP2 was shown to start at Val37 [22], which would predict that mature MTB ClpP2 starts with the Ile15 residue (Figure 3).

Several truncated variants of ClpP1 starting with Met7 (M7ClpP1) or Ser9 (S9ClpP1), and of ClpP2 starting with Arg13 (ClpP2R13), Tyr14 (ClpP2Y14), Ile15 (ClpP2I15), or Leu16 (ClpP2L16) residues were produced and purified in SG1146a cells (see additional file 4), and analyzed for peptide cleavage and oligomeric assembly.

**Figure 4:**
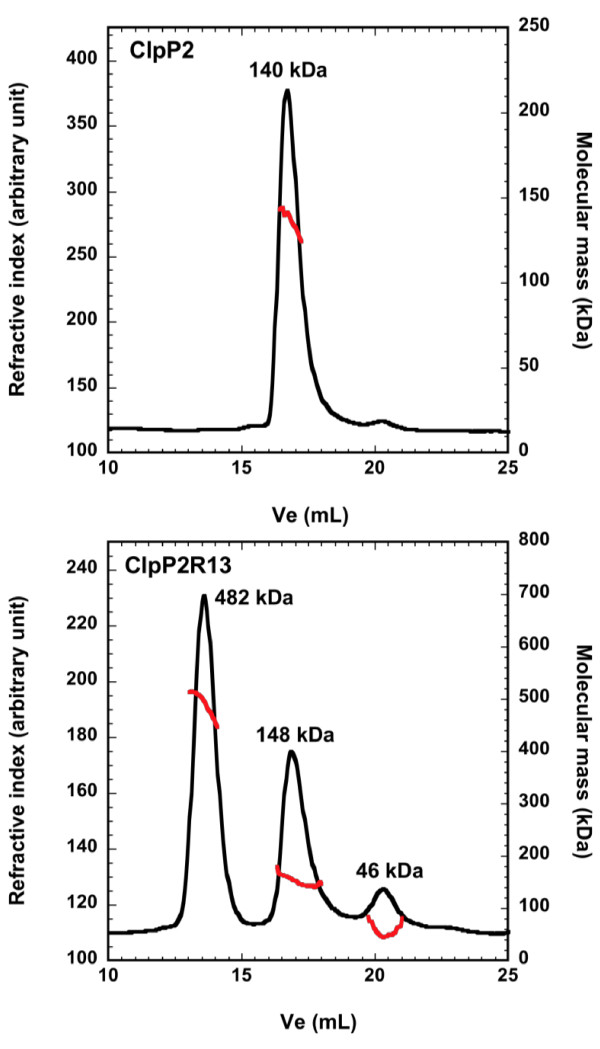
**Effect of removing putative propeptide on the assembly of ClpP2**. 200 μg of purified ClpP2 (upper panel) or of the ClpP2R13 variant (lower panel) were subjected to a size exclusion chromatography as followed by a triple detector array as described in the Methods section. The refractive index (black line) and the molecular mass (red line) were plotted as a function of the elution volume. Average molecular weights obtained by static light scattering are indicated at the top of each peak.

Removing the putative prosequences of ClpP1 did not change its elution during SEC (data not shown). Likewise, most of the processed ClpP2 variants eluted with an elution volume comparable to that of wild-type ClpP2 (data not shown). However, the ClpP2R13 variant surprisingly eluted as three main species. In order to determine precisely the nature of these different oligomeric species, full-length ClpP2 and the ClpP2R13 variant were subjected to size-exclusion chromatography coupled online to a triple detector array (SEC-TDA) and further analyzed by static light scattering measurements. In SEC-TDA experiment, the analysis of full-length ClpP2 peak indicated the presence of a range of assemblies with a weight average molecular mass of 140 kDa compatible with hexamers or heptamers of ClpP2 subunits (Figure 4, upper panel). Analysis of the ClpP2R13 variant by SEC-TDA indicated species with molecular masses of 482.5 kDa, 148.0 kDa and 46.3 kDa corresponding to 21-mer, heptamer, and dimer species respectively (Figure 4, lower panel). The 21-mer oligomeric species was also observed during the elution of the ClpP2Y14, but in a lower amount (data not shown). This high molecular weight complex is reminiscent of that observed with *E. coli *ClpP in the absence of salt [19] and could correspond to three stacked heptameric rings. Therefore, removing the putative prosequence of ClpP2 did promote the formation of higher order oligomers but not of the expected tetradecamer. None of those ClpP1 or ClpP2 variants exhibited any activity toward Suc-LY-Amc or Boc-LRR-Amc peptides (data not shown). Thus, removing putative prosequences was not sufficient for allowing ClpP1 and ClpP2 to assemble into tetradecamers with activity toward model peptides.

### Deleting the entry gate does not activate ClpP1 and ClpP2

Since the N-terminal extremity of ClpP2 influenced its oligomerisation state, we tested whether further deletions in their N-termini would favor assembly of ClpP1 and ClpP2 in tetradecamer.

Recent studies have demonstrated a gating mechanism for *E. coli *ClpP comparable to that of proteasomes, a self-compartmentalized protease with a similar barrel-shaped architecture. In general, the first 20 residues of mature ClpP (after its processing) adopt a flexible conformation that can explain why they are not always visible in electron density map. In the mature processed ClpP, the N-terminal extremity can adopt different secondary structures and therefore modulate the size of the entry pore [23]. In the "up" conformation, a portion of the N-terminus extends outwards the access pore whereas in the "down" conformation it is located within the axial pore. For *E. coli *ClpP, removing the residues involved in the gating mechanism allowed degradation of unfolded substrates in the absence of the ATPase complex [24]. For the eukaryotic 20S proteasomes, the entry gate precludes entry of even small size peptides [25].

**Figure 5:**
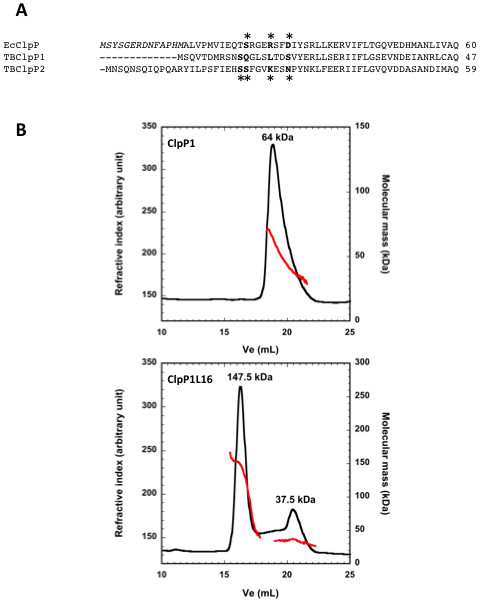
**Effect of removing the putative gate on the assembly of mature ClpP1**. (A) The N-terminal sequences of MTB ClpP1 (H37Rv strain, gi: 41353667) and ClpP2 (H37Rv strain, gi: 2791500) were aligned with that of *E. coli *ClpP (gi: 89107307). The propeptide of *E. coli *ClpP is written in italics. The first residues of deletion variants of *E. coli *ClpP are indicated in bold and by an asterisk above the sequence. The corresponding residues in MTB ClpP1 and ClpP2 sequences are written in bold and indicated with an asterisk below their sequences. (B) 200 μg of purified ClpP1 (upper panel) or of the ClpP1L16 variant (lower panel) were subjected to a size exclusion chromatography as followed by a triple detector array as described above.

In MTB ClpP1, the residues Ser15-Glu27 fold as a helix that is longer than the corresponding helix in ClpP orthologues and that protruded into the axial pore of the tetradecamer, making a gating mechanism likely [21]. To see whether removing the putative gate in MTB ClpP1 and ClpP2 would have an effect on peptide cleavage, N-terminal gate deletion variants were constructed based on the alignment with *E. coli *ClpP (Figure 5A). In *E. coli*, deletion of the first 10, 14, and 17 first amino acids (based on the numbering of mature ClpP) activated degradation of unfolded substrates to a different extent, with the Δ 14ClpP variant being the most active. Corresponding deletion variants were produced and their peptidase activity and tetradecameric assembly were tested. None of them exhibited any significant peptidase activity toward the Suc-LY-Amc peptide (data not shown), suggesting that the absence of chymotryptic activity toward this peptide in WT ClpP1 and ClpP2 was not due to any obstruction of the pore entrance by their N-termini.

When we examined their assembly by SEC-TDA, we found that when the first 15 residues of ClpP1 were absent, an additional peak was detected. Indeed, full-length ClpP1 exhibited the presence of a several assemblies with molecular masses ranging from 30 to 70.5 kDa compatible with complexes varying from monomers to trimers of ClpP1 subunits (Figure 5B, upper panel). The ClpP1L16 variant eluted as a sharper peak corresponding to a species having a molecular mass complex of about 150 kDa, compatible with ClpP1 heptamers (Figure 5B, lower panel). Thus, removing the N-terminal extremity of ClpP1 likely favors assembly of the ClpP1 heptamericring.

Deleting the amino terminal residues of ClpP2 slightly decreased its elution volume during SEC but did not promote higher order oligomeric assembly above heptameric form (data not shown). These finding indicated that further removing the N-terminal extremities of ClpP1 and ClpP2 could stabilize the heptamericring.

### Coproduction of the peptidases with the ATPases ClpC1 and ClpX

Most of ClpPs, when tested, are able to degrade small peptides in the absence of the ATPase complex. One exception is the human mitochondrial ClpP that required the ATPase ClpX to assemble into stable tetradecamer with a high peptidase activity [26]. In the MTB genome, three genes have been annotated as Clp/hsp100 proteolysis associated ATPase, *clpC1 *(Rv3596c), *clpC2 *(Rv2667), and *clpX *(Rv2457c) (Pasteur Institute TubercuList, http://genolist.pasteur.fr/TubercuList).

**Figure 6:**
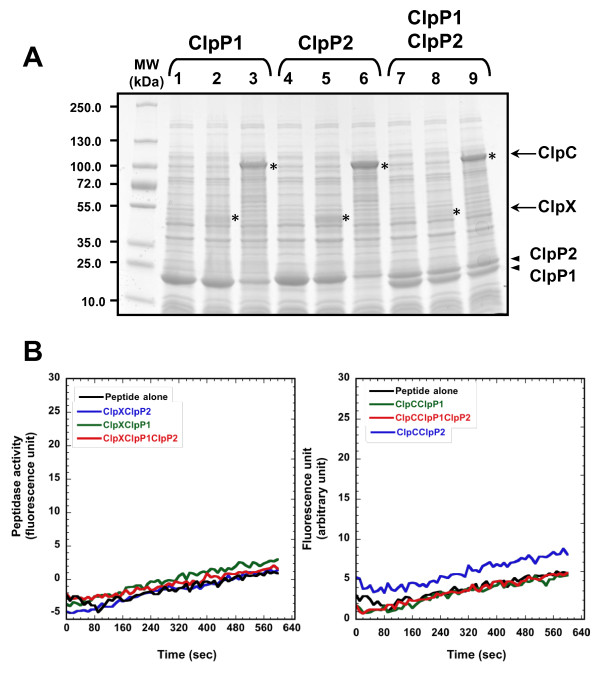
**Coproducing ClpP1 and ClpP2 with ClpX and ClpC ATPases did not result in Suc-LY-Amc cleavage**. (A) 10 μg of total extract of cells producing ClpP1 (lane 1), ClpP1 and ClpX (lane 2), ClpP1 and ClpC (lane 3), ClpP2 (lane 4), ClpP2 and ClpX (lane5), ClpP2 and ClpC (lane 6), ClpP1 and ClpP2 without (lane 7) or with ClpX (lane 8) or ClpC (lane 9) were loaded on a 4-15% gradient SDS-PAGE stained with coomassie blue. The electrophoretic mobilities of ClpX and ClpC are indicated by asterisks. The molecular masses of the markers were indicated on the left in kDa. (B) Hydrolysis of the Suc-LY-Amc peptide in the absence (black trace) or in the presence of 10 μg of total extracts of SG1146a cells overexpressing *clpP1(his)_6 _*and *clpX *(left panel, green trace), *clpP2(his)_6 _*and *clpX *(left panel, blue trace), the *clpP1-clpP2(his)_6 _*operon together with *clpX *(left panel, red trace), *clpP1(his)_6 _*and *clpC *(right panel, green trace), *clpP2(his)_6 _*and *clpC *(right panel, blue trace), the *clpP1-clpP2(his)_6 _*operon together with *clpC *(right panel, red trace).

Primary sequences of ClpC1 and ClpX contain the typical Clp ATPase features, *i. e*. one or two AAA (ATPase Associated with various cellular Activities) domains. However, ClpC2 protein does not exhibit any AAA or other ATPase motif and the purified ClpC2 did not have any ATPase activity (unpublished data). The presence of two ClpN domains in the ClpC2 sequence might have been at the origin of the mistaken annotation of this open reading frame as a Clp ATPase. Therefore, only ClpC1 and ClpX seem to be putative ATPase partners for ClpP1 and ClpP2 peptidases. To investigate whether MTB ClpP1 and ClpP2 require their ATPase partners to hydrolyze peptides, ClpP1 and ClpP2 were produced with ClpC or ClpX (Figure 6A) and peptide cleavage was tested in total extracts. The presence of ClpX or ClpC did not result in Suc-LY-Amc hydrolysis (Figure 6B). Therefore, the absence of Suc-LY-Amc cleavage by ClpP1 and ClpP2 was not due to a requirement for the ATPase complex.

### ClpP1 is responsible for ClpP1 and ClpP2 processing

**Figure 7:**
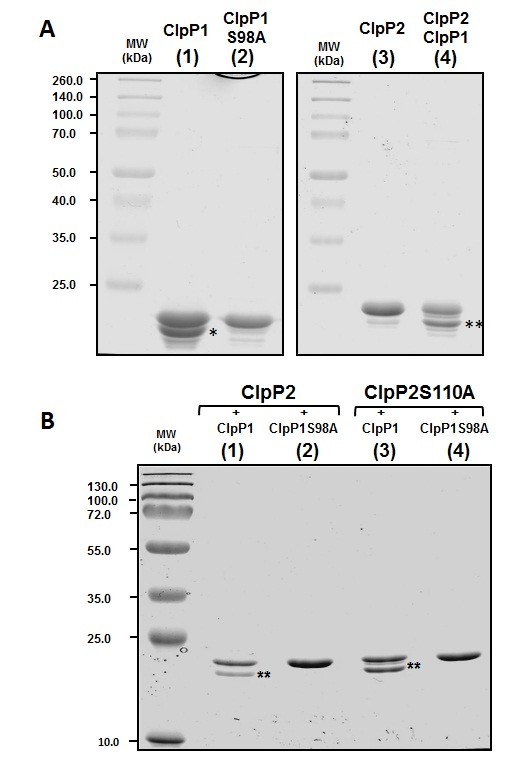
**Proteolytic processing of ClpP1 and ClpP2 by ClpP1**. (A) 2.5 μg of pure ClpP1 (lane 1), of ClpP1S98A variant (lane 2), of pure ClpP2 (lane 3), and 4.0 μg of purified ClpP2 produced in the presence of ClpP1 (lane 4) were loaded on a 15% SDS-PAGE stained with Coomassie blue. The molecular masses of the markers were indicated on the left in kDa. (B) 2.0 μg of purified ClpP2 produced in the presence of ClpP1 (lane 1) or in the presence of ClpP1S98A (lane 2), and 2.0 μg of purified ClpP2S110A purified in the presence of ClpP1 (lane 3) or in the presence of ClpP1S98A (lane 4) were loaded on a 15% SDS-PAGE stained with Coomassie blue. The molecular masses of the markers were indicated on the left in kDa.

When produced in *E. coli*, ClpP1 migrated as two bands on SDS-PAGE (Figure 7A, lane 1). Microsequencing revealed that the upper band contained a protein starting with SQVTDM, corresponding to a full-length ClpP1 that had its initiating methionine residue removed. The smaller protein in ClpP1 preparation (labeled * in Figure 7A) started with SNSQG, corresponding to ClpP1 starting at the Ser9 residue. When the Ser catalytic residue of ClpP1 was replaced by an Ala (Ser98Ala variant), this form of ClpP1 was not visible (Figure 7A, lane 2). Therefore, proteolytic activity of ClpP1 was responsible for its own cleavage.

ClpP2 migrated mainly as a single band on SDS-PAGE (Figure 7A, lane 3). Microsequencing showed that it corresponded to the full-length ClpP2 (starting with MNSQNS residues). When ClpP2 was coproduced with ClpP1, two bands were obtained (Figure 7A, lane 4). The protein of the upper band started with MNSQNS residues and corresponded to full-length ClpP2. The protein in the lower band (labeled ** in Figure 7A and 7B) started with RYILP and corresponded to ClpP2 starting at the Arg13 residue. Therefore, coproducing ClpP1 and ClpP2 led to processing of ClpP2. In order to test which peptidase was responsible for ClpP2 processing, we inactivated ClpP1 and ClpP2 by replacing their active site serine by an alanine residue (ClpP1Ser98Ala and ClpP2Ser110Ala) in the *clpP1-clpP2(his)_6 _*operon. Inactivating ClpP2 only did not prevent its processing (Figure 7B, lane 3) whereas ClpP2 processing was not observed when ClpP1 was inactivated (Figure 7B, lane 2). Altogether, these results demonstrated that recombinant ClpP1 possessed a proteolytic activity responsible for its own cleavage after the Arg8 residue and for ClpP2 cleavage after the Ala12 residue. It is noteworthy that the truncated ClpP1 form produced by the proteolytic activity of ClpP1 corresponded to the putative processed ClpP1 as predicted by sequence alignment. In ClpP2, the processing cleavage site catalyzed by ClpP1 was in the vicinity of that predicted by sequence alignment. In conclusion, even though no Suc-LY-amc cleavage was observed by ClpP1, this peptidase exhibited the ability to hydrolyze a peptide bond.

## Discussion

Most of our knowledge on the mechanism of protein degradation by ClpP protease is based on studies of *E. coli *ClpP. This peptidase is able alone to hydrolyze the model dipeptide Suc-LY-Amc *in vitro *[5]. In this study, we have shown that, in conditions that normally allow Suc-LY-Amc cleavage by *E. coli *ClpP, MTB ClpP1 and ClpP2 do not hydrolyze this model peptide. However, a proteolytic activity was uncovered for ClpP1, ruling out the possibility that, at least for ClpP1, those peptidases were produced in *E. coli *as totally inactive.

An absence of peptidase activity was not due to the presence of a His tag at the C-termini of ClpP1 or ClpP2 since purifying those peptidases without any tag did not allow hydrolysis of Suc-LY-Amc or of other model peptides, nor it promoted correct tetradecameric assembly.

A failure to detect model peptide cleavage was not due to an obstruction of the entry pore by the N-terminal extremities of ClpP1 and ClpP2, predicted to be highly flexible, since removing these extremities did not result in peptide cleavage. Furthermore, coproducing the ClpX and ClpC ATPase complexes with ClpP1 and ClpP2 did not allow Suc-LY-Amc cleavage, showing that an absence of peptide model hydrolysis by mycobacterial ClpPs was not due to an obligatory requirement for the ATPases.

One plausible explanation for an absence of Suc-LY-Amc cleavage would be that mycobacterial ClpPs require different physico-chemical conditions to hydrolyze this peptide than those needed by *E. coli *ClpP. In fact, based on crystal structure determination, the tetradecamer of ClpPs from different organisms could be grouped into two structural states: an extended state fully active toward the Suc-LY-Amc model peptide (seen with *E. coli*, *Homo sapiens*, *H. pylori *ClpPs) and a more compact state that likely corresponds to an inactive state (seen with *M. tuberculosis*, *S. pneumoniae*, *Plasmodium falciparum*) [27]. Recombinant ClpP1 and ClpP2 might be isolated in the compact state and require specific physico-chemical conditions to switch to the extended fully active conformation toward model peptides.

Also, the specificities of peptide bond hydrolysis could be different than those of *E*. *coli *ClpP. Indeed, the nature of the amino acid in the P1 position relative to the scissile peptide is important in controlling the hydrolysis rate. For instance, *E. coli *ClpP has been shown to exhibit the greatest degradation rate when a large aromatic amino acid residue is in the P1 position (as in the Suc-LY-Amc peptide) [28].

However, this feature is not shared by all ClpPs since *P. falciparum *ClpP exhibited a preference for an Arg residue in P1 position [29]. Likewise, MTB ClpP1 and ClpP2 could require another amino acid residue at the P1 position. Consistent with such a hypothesis is our finding that ClpP1 can cleave a peptide bond after Ala and Arg residues. In the light of these findings, short peptides of those specificities (H-A-Amc, Suc-AAA-Amc, H-GR-Amc, Boc-LRR-Amc, Bz-VGR-Amc) were tested but none of them were cleaved by ClpP1 or ClpP2. Determination of the peptide specificities of MTB ClpPs must await further studies.

In this study, we have also shown that recombinant MTB ClpP1 and ClpP2 could not assemble as tetradecamers in solution. Determining the X-ray structure of ClpP1 has shown that ClpP1 could assemble in a tetradecamer under the crystal conditions but the interactions between the two heptamers that stabilize the tetradecamer were weaker than those in other ClpPs; and ClpP1 was mainly isolated as a heptamer in solution [21]. Our findings suggest that a weak interaction between heptamers could also apply for ClpP2 assembly. In our study, we showed that deleting the amino-terminal extremity of ClpP1 and ClpP2 favored a higher order assembly. In most of the deposited X-ray structures, the amino-terminal extremity of mature ClpP is unmodelled because non interpretable in the electron density. This suggested a high flexibility in this portion of the protein. Indeed, Bewley et al. [23] demonstrated that the first 20 residues in the mature *E. coli *ClpP could adopt two different conformations. In the X-ray structures of the unprocessed full length MTB ClpP1, the first 15 residues were not visible and the Ser15-Glu27 portion formed a α-helix longer than in other orthologous ClpPs that partially occupies the axial pore [21]. The flexibility of this might hinder a correct assembly of mycobacterial ClpPs and a deviation in its conformation form other orthologous ClpPs might explain a difference in the stabilization of the tetradecamer. Whether an interaction of mycobacterial ClpPs with the ATPase ClpX and ClpC could stabilize a tetradecameric assembly remains to be tested.

Despite that recombinant ClpP1 did not assemble in a tetradecamer, it exhibited a proteolytic activity that was reminiscent of the autocatalytic processing of ClpP proteases. Autocatalytic processing in ClpP might not require the tetradecameric functional assembly required for the processive proteolytic activity of ClpP. Indeed, the minimal functional structure for the hydrolytic activity of ClpP was found to be a single heptameric ring [30].

## Conclusion

In this study, we have uncovered an unconventional mechanism of oligomeric assembly and proteolytic activity for MTB ClpP1 and ClpP2 that are distinct from those of other ClpPs, especially the well-characterized *E. coli *ClpP. A better knowledge of such ATP-dependent proteases which are potentially important for the survival and virulence of MTB could offer new hope in the need for developing new drug to treat tuberculosis.

## Methods

### Bacterial strain and primers

*E. coli *BL21(DE3) [31] (Novagen) and BL21(DE3) *ClpP*::*cam *(SG1146a) (Susan Gottesman) strains were used to express recombinant proteins. All primers used are listed in the additional file 5.

### Cloning of *clpP2 *(Rv2460c) and *clpP1 *(Rv2461c) ORFs

*clpP1 *ORF (Rv2461c) was amplified by PCR and cloned into the pET26b vector (Novagen) using the ClpP1-5Nde and ClpP1-3Xho primers (see additional file 5) at the NdeI and XhoI sites, giving rise to the pNB71 plasmid containing the *clpP1 *ORF with a C-terminal (His)_6 _tag. *clpP2 *ORF (Rv2460c) was amplified by PCR using the ClpP2-5Nde and ClpP2-3HindChis primers and cloned at the NdeI and HindIII sites into the pet26b vector, giving rise to the pNB74 plasmid in which the *clpP2 *ORF was C-terminal (His)_6 _tagged.

The plasmid expressing the *clpP1-clpP2(his)_6 _*operon was constructed by first amplifying the *clpP1 *ORF with the ClpP1-5Nde and ClpP1-3Hind primers and cloned at the NdeI and HindIII sites into the pET26b vector. The resulting plasmid was named pNB70. The *clpP2(his)_6 _*ORF was amplified by PCR from the pNB74 plasmid using the rbsClpP2Hind and ClpP2-3Not primers and introduced at the HindIII and NotI sites into the pNB70 plasmid. The resulting plasmid was named pNB82. *clpP1 *C-terminal (His)_6 _tagged ORF starting at the Met7, Ser9, Ser11, Gln12, Leu16, and Ser 19 residues were obtained by PCR amplification using the ClpP1M7, ClpP1S9, ClpP1S11, ClpP1Q12, ClpP1L16 and ClpP1S19 primers respectively as well as the ClpP1-3Xho primer and introduced at the NdeI and XhoI sites into the pET26b vectors. *clpP2 *C-terminal (His)_6 _tagged ORF starting at the Arg13, Tyr14, Ile15, Leu16, Ser 23, Ser24, Lys28, and Asn 31 residues were obtained by PCR amplification using the ClpP2R13, ClpP2Y14, ClpP2I15, ClpP2L16, ClpP2S23, ClpP2S24, ClpP2K28, and ClpP2N31 primers respectively as well as the ClpP2-CHis primer and introduced at the NdeI and HindIII sites into the pET26b vector. DNA sequence of all open reading frames was checked by DNA sequencing (Beckman Coulter Genomics).

### Protein production and purification

Cells were cultivated at 30°C in Luria-Bertani (LB) medium supplemented when necessary with 30 μg/mL of kanamycin and 25 μg/mL of chloramphenicol. BL21(DE3) or SG1146a cells were transformed with an expression vector carrying the *clpP1 *and/or *clpP2 *ORFs and cultivated until an OD_600 _of 0.6. Protein expression was then induced with 1 mM isopropyl-β-D-thiogalactopyranoside (IPTG). After centrifugation, cell pellets were resuspended in the buffer A (50 mM NaH_2_PO4 pH 8.0, 300 mM NaCl, 10 mM Imidazole, 10% glycerol) and lysed by sonication. The soluble fraction obtained after a 60 minutes centrifugation at 40,000 g at 4°C was loaded on nickel-nitrilotriacetic (Ni-NTA) resin (Qiagen) and the resin was washed with buffer A. His-tagged proteins were eluted with buffer B (50 mM NaH_2_PO4 pH 8.0, 300 mM NaCl, 250 mM Imidazole, 10% glycerol). After elution, His-tagged proteins were dialyzed against 50 mM Tris-HCl pH 7.5, 200 mM KCl, 2 mM DTT, 0.1 mM EDTA, 10% glycerol and concentrated. Protein concentrations were determined with Coomassie Plus Protein Assay Reagent (Thermo Scientific).

### Inactivation of ClpP1 and ClpP2

The active site Ser residues in ClpP1 (Ser98) and in ClpP2 (Ser110) were replaced by an Ala residue using the Quick Change Multi Site-Directed Mutagenesis kit (Stratagene) according to the manufacturer's recommendation with the ClpP1S98A and ClpP2S110A primers respectively. ClpP1 and ClpP2 variants were produced and purified in SG1146a cells as described above.

### Peptide hydrolysis

1 mM of fluorogenic Suc-LY-Amc peptide diluted in 100% DMSO was incubated with purified ClpP1 or ClpP2 or with total extracts as indicated at 37°C in 50 mM Tris pH 7.5, 100 mM KCl and 1 mM DTT. Hydrolysis of the peptide was followed by measuring the release of amc (7-amino-4-methylcoumarin) in a spectrofluorometer (λ_ex _380 nm; λ_em _460 nm).

### Size exclusion chromatography

SEC was carried out at room temperature on a Superdex 200 10/30 column (GE Healthcare) equilibrated with 25 mM Tris-HCl, pH 7.5, 150 mM KCl, and 1 mM DTT. Elution was performed using the same buffer at a flow rate of 0.5 mL/min, and absorbance was measured at 280 nm. The column was calibrated with Ferritin (440 kDa), Catalase (232 kDa), Aldolase (158 kDa), Bovin Serum Albumin (67 kDa), Ovalbumin (43 kDa), Chymotrypsinogen (25 kDa), and Ribonuclease (17.3 kDa).

For the SEC-TDA, samples were separated according to their hydrodynamic size on the same Superdex 200 column in 25 mM Tris-HCl, pH 7.5, 150 mM KCl, and 1 mM β-mercaptoethanol at 20°C. Molecular masses of the eluted sample were determined online on a TDA model 302 (Malvern Instruments Ltd, UK) at 20°C. The TDA contains 4 detectors in line: (i) a static light-scattering cell with two photodiode detectors at 7° for low-angle light scattering (LALS) and at 90° for RALS, (ii) a deflection refractometer, (iii) a photometer, and (iv) a differential viscometer. BSA was used for molecular mass calibration, and all data were acquired and processed using the Omnisec software (Viscotek Ltd.) as described elsewhere [32].

### Coproduction of ClpP1 and ClpP2 with the ClpC and ClpX ATPases

The *clpX *ORF (Rv2457c) was PCR amplified using the ClpX-5Nco and ClpX-3Hind primers and introduced at the NcoI and HindIII sites into the pCDFDuet vector (Novagen). The resulting plasmid was named pNB104. The *clpP1 *ORF was amplified using the ClpP1-5Nde and ClpP1-3Pac primers and introduced at the NdeI and PacI in the pNB104 plasmid. The final plasmid (pNB106) contained the *clpX *and *clpP1(his)_6 _*under the control of two independent T7 promotors.

In order to express *clpP2 *together with *ClpX*, the *clpP2 *ORF was first amplified with the ClpP2-5Nco and ClpP2-CHis primers and cloned into the pCDFDuet vector at the NcoI and HindIII sites. The resulting plasmid was named pNB79. The *clpX *ORF was amplified with the ClpX-5Nde and ClpX-3Pac primers and introduced into the pNB79 plasmid at the NdeI and PacI sites. The resulting plasmid (pNB107) contained the *clpP2(his)_6 _*and the *clpX *ORF under two independent T7 promotors.

The *clpC *ORF (Rv3596c) was PCR amplified using the ClpC-5Nco and ClpC-3Hind primers and introduced at the NcoI and HindIII sites into the pCDFDuet vector. The resulting plasmid was named pNB108. Then, the *clpP1 *ORF introduced at the NdeI and PacI in the pNB108 plasmid as described for the pNB106 plasmid. The final plasmid (pNB110) contained the *clpC *and *clpP1(his)_6 _*under the control of two independent T7 promotors.

In order to express *clpP2 *together with *clpC*, the *clpC *ORF was first amplified with the ClpC-5Nde and ClpC-Pac primers and cloned into the pNB79 plasmid at the NdeI and PacI sites. The resulting plasmid was named pNB111 and it contained the *clpP2(his)_6 _*and the *clpX *ORF under two independent T7 promotors.

SG1146a cells were transformed with the pNB104, pNB106, pNB107, pNB108, pNB110, pNB111 plasmids with or without pNB82 as indicated in the legends. Cells were cultivated at 30°C in LB medium complemented when necessary with 30 μg/mL of kanamycin, 50 μg/mL of streptomycin and 25 μg/mL of chloramphenicol. Protein production was induced with IPTG as described above and cells were harvested. Cells pellets were resuspended in 50 mM Tris-HCl pH 7.5, 200 mM KCl, 2 mM DTT, 0.1 mM EDTA, 10% glycerol and lyzed by sonication. Peptide hydrolysis was measured in 50 mM Tris pH 7.5, 100 mM KCl and 1 mM DTT, 10 mM MgCl_2_, 4 mM ATP and 0.02% Triton X100 as described above.

## List of abbreviation used

Clp: caseinolytic protease; ORF: open reading frame; MTB: *Mycobacterium tuberculosis*; Suc-LY-Amc: N-Succinyl-Leu-Tyr-7 amido 4 methylcoumarin; Bz-VGR-Amc: benzoyl-Val-Gly-Arg-7 amido 4 methylcoumarin; Boc-LRR-Amc: terbutyloxycarbonyl-Leu-Arg-Arg-7 amido 4 methylcoumarin; SEC: size-exclusion chromatography; SEC-TDA: size-exclusion chromatography coupled online to a triple detector array; DTT: dithiothreitol; ATP: adenosine triphosphate; IPTG: isopropyl-β-D-thiogalactopyranoside.

## Authors' contributions

NB conceived, designed and performed experiments, analyzed data and wrote the manuscript. BR carried out and analyzed Sec-TDA experiments. MM helped in purifying and characterizing ClpP1 and ClpP2. MO-L helped in purifying processed ClpP1 and ClpP2. All read and approved the final manuscript.

## Supplementary Material

Additional file 1 **Figure S1: Sequence alignment of MTB ClpP1 and ClpP2 with that of *E. coli *ClpP**. The sequences of MTB ClpP1 (H37Rv strain, gi: 41353667) and ClpP2 (H37Rv strain, gi: 2791500) were aligned with that of *E. coli *ClpP (gi: 89107307) using ClustalW program http://www.ebi.ac.uk/Tools/msa/clustalw2. The propeptide of *E. coli *ClpP is written in bold. The residues in the catalytic triad (Ser, His, Asp) are indicated in bold and underlined. Identical (*), the similar (.), and very similar (:) residues are indicated below the sequences. Click here for file

Additional file 2 **Figure S2: Peptidase activity of *E. coli *ClpP copurified with ClpP1 and ClpP2**. Hydrolysis of 1 mM Suc-LY-Amc peptide in the presence of 10 μg of Ni^2+ ^column-purified proteins from BL21(DE3) cells overexpressing the pET26b plasmid (black triangles), BL21(DE3) cells overexpressing the *clpP1-clpP2(his)_6 _*operon (black squares), or SG1146a cells overexpressing the *clpP1-clpP2(his)_6 _*operon (black circles) after 50 days of storage of the protein preparation at 4°C. Hydrolysis of the peptide was followed by measuring the release of amc (7-amino-4-methylcoumarin) in a spectrofluorometer (λ_ex _380 nm; λ_em _460 nm). Click here for file

Additional file 3 **Figure S3: Interaction of *E. coli *ClpP with ClpP1 or ClpP2**. Soluble extracts were prepared from SG1146a cells producing untagged *E. coli *ClpP alone or together with ClpP1(His)_6 _or ClpP2(His)_6 _and loaded on a Ni^2+ ^column. After extensive washing with buffer A (50 mM NaH_2_PO4 pH 8.0, 300 mM NaCl, 10 mM Imidazole, 10% glycerol), resin-bound proteins were eluted with buffer B (50 mM NaH_2_PO4 pH 8.0, 300 mM NaCl, 250 mM Imidazole, 10% glycerol). The presence of *E. coli *ClpP was analyzed by a 15% SDS-PAGE and detected by immunoblot using an anti ClpP antibody that exhibited cross-reaction with ClpP1 and ClpP2. **(A) **The indicated samples were loaded on a 15% SDS-PAGE stained with Coomassie blue. The molecular mass markers are indicated on the left. Lanes 1-3: 10 μg of the soluble extract of SG1146a cells producing untagged *E. coli *ClpP (lane 1), ClpP1(His)_6 _(lane 2), or untagged *E. coli *ClpP together with ClpP1(His)_6 _(lane 3) that were loaded on the Ni^2+ ^column. Lanes 4-6: proteins eluted from Ni^2+ ^column when SG1146a cells produced untagged *E. coli *ClpP (lane 4), ClpP1(His)_6 _(lane 5), or untagged *E. coli *ClpP together with ClpP1(His)_6 _(lane 6). The upper band in lane 6 is ClpP1(His)_6 _as determined by anti His tag immunoblot (data not shown) and the lower band is *E. coli *ClpP as determined by anti ClpP immunodetection in the panel (C). **(B) **The indicated samples were loaded on a 15% SDS-PAGE stained with Coomassie blue. The molecular mass markers are indicated on the left. Lanes 1-3: 10 μg of the soluble extract of SG1146a cells producing untagged *E. coli *ClpP (lane 1), ClpP2(His)_6 _(lane 2), or untagged *E. coli *ClpP together with ClpP2(His)_6 _(lane 3) that were loaded on the Ni^2+ ^column. Lanes 4-6: proteins eluted from Ni^2+ ^column when SG1146a cells produced untagged *E. coli *ClpP (lane 4), ClpP2(His)_6 _(lane 5), or untagged *E. coli *ClpP together with ClpP2(His)_6 _(lane 6). The upper band in lane 6 is ClpP2(His)_6 _as determined by anti His tag immunoblot (data not shown) and the lower band is *E. coli *ClpP as determined by anti ClpP immunodetection in the panel (C). **(C) **Immunodetection of ClpP proteins in 2 μg of soluble extract of SG1146a cells producing *E. coli *ClpP (lane 1) and in the sample eluted from the Ni^2+ ^column when the SG1146a cells produced untagged *E. coli *ClpP (lane 2), ClpP1(His)_6 _(lane 3), ClpP2(His)_6 _(lane 4), untagged *E. coli *ClpP together with ClpP1(His)_6 _(lane 5), or untagged *E. coli *ClpP together with ClpP2(His)_6 _(lane 6). The proteins were separated on a 15% SDS-PAGE and transferred onto nitrocellulose. The ClpP proteins were detected using an anti ClpP antibody that interacted with MTB ClpP1 and ClpP2 as well as with *E. coli *ClpP. Experimental evidence of an interaction between *E. coli *ClpP and MTB ClpP1 or ClpP2 was also observed by producing *E. coli *ClpP(His)_6 _together with S-tagged ClpP1 or ClpP2 (data not shown). Click here for file

Additional file 4 **Figure S4: Purified ClpP1 and ClpP2 variants**. About 5 μg of the indicated purified proteins were loaded on a 12% SDS-PAGE stained with Coomassie blue. The molecular mass markers are indicated on the left. **(A) **Full length ClpP1 (M1) and the variants starting at the Met7 (M7) and Ser9 (S9). **(B) **Full length ClpP2 (M1) and the variants starting at Arg13 (R13), Tyr14 (Y14), Ile15 (I15), Leu16 (L16). **(C) **Full length ClpP1 (M1) and the variants starting at the Ser11 (S11), Gln12 (Q12), Leu16 (L16), and Ser19 (S19). **(D) **Full length ClpP2 (M1) and the variants starting at the Ser23 (S23), Ser24 (S24), Lys28 (K28), and Asn31 (N31). Click here for file

Additional file 5 **Table S1: Oligonucleotides used in this study**. Click here for file
