# Supplementary material for: Assembly and proteolytic processing of mycobacterial ClpP1 and ClpP2
Source: BMC Biochem. 2011 Dec 1;12:61. doi: 10.1186/1471-2091-12-61 (PMC3258218; doi:10.1186/1471-2091-12-61)
Supplement: Additional file 1 — Figure S1: Sequence alignment of MTB ClpP1 and ClpP2 with that of E. coli ClpP. The sequences of MTB ClpP1 (H37Rv strain, gi: 41353667) and ClpP2 (H37Rv strain, gi: 2791500) were aligned with that of E. coli ClpP (gi: 89107307) using ClustalW program http://www.ebi.ac.uk/Tools/msa/clustalw2. The propeptide of E. coli ClpP is written in bold. The residues in the catalytic triad (Ser, His, Asp) are indicated in bold and underlined. Identical (*), the similar (.), and very similar (:) residues are indicated below the sequences. [file 1471-2091-12-61-S1.PPT]

## Slide 1
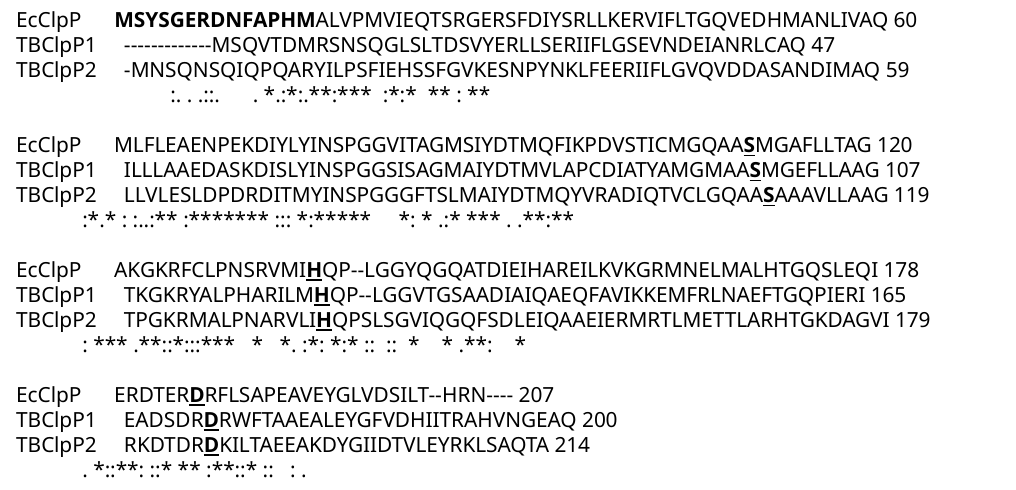

EcClpP MSYSGERDNFAPHMALVPMVIEQTSRGERSFDIYSRLLKERVIFLTGQVEDHMANLIVAQ 60
TBClpP1 -------------MSQVTDMRSNSQGLSLTDSVYERLLSERIIFLGSEVNDEIANRLCAQ 47
TBClpP2 -MNSQNSQIQPQARYILPSFIEHSSFGVKESNPYNKLFEERIIFLGVQVDDASANDIMAQ 59
 :. . .::. . *.:*:.**:*** :*:* ** : **
EcClpP MLFLEAENPEKDIYLYINSPGGVITAGMSIYDTMQFIKPDVSTICMGQAASMGAFLLTAG 120
TBClpP1 ILLLAAEDASKDISLYINSPGGSISAGMAIYDTMVLAPCDIATYAMGMAASMGEFLLAAG 107
TBClpP2 LLVLESLDPDRDITMYINSPGGGFTSLMAIYDTMQYVRADIQTVCLGQAASAAAVLLAAG 119
 :*.* : :..:** :******* ::: *:***** *: * .:* *** . .**:**
EcClpP AKGKRFCLPNSRVMIHQP--LGGYQGQATDIEIHAREILKVKGRMNELMALHTGQSLEQI 178
TBClpP1 TKGKRYALPHARILMHQP--LGGVTGSAADIAIQAEQFAVIKKEMFRLNAEFTGQPIERI 165
TBClpP2 TPGKRMALPNARVLIHQPSLSGVIQGQFSDLEIQAAEIERMRTLMETTLARHTGKDAGVI 179
 : *** .**::*:::*** * *. :*: *:* :: :: * * .**: *
EcClpP ERDTERDRFLSAPEAVEYGLVDSILT--HRN---- 207
TBClpP1 EADSDRDRWFTAAEALEYGFVDHIITRAHVNGEAQ 200
TBClpP2 RKDTDRDKILTAEEAKDYGIIDTVLEYRKLSAQTA 214
 . *::**: ::* ** :**::* :: : .
