# Supplementary figures and images for: Assembly and proteolytic processing of mycobacterial ClpP1 and ClpP2
Source: BMC Biochem. 2011 Dec 1;12:61. doi: 10.1186/1471-2091-12-61 (PMC3258218; doi:10.1186/1471-2091-12-61)

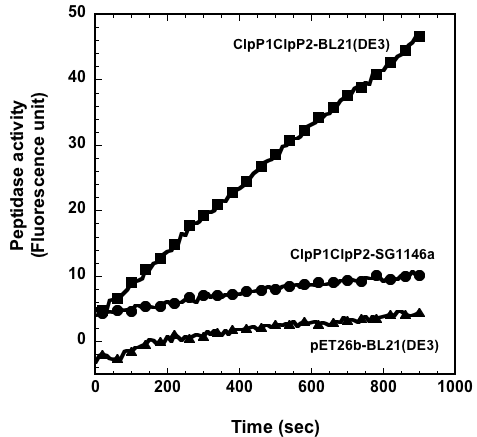

Supplement: Additional file 2 — Figure S2: Peptidase activity of E. coli ClpP copurified with ClpP1 and ClpP2. Hydrolysis of 1 mM Suc-LY-Amc peptide in the presence of 10 μg of Ni2+ column-purified proteins from BL21(DE3) cells overexpressing the pET26b plasmid (black triangles), BL21(DE3) cells overexpressing the clpP1-clpP2(his)6 operon (black squares), or SG1146a cells overexpressing the clpP1-clpP2(his)6 operon (black circles) after 50 days of storage of the protein preparation at 4°C. Hydrolysis of the peptide was followed by measuring the release of amc (7-amino-4-methylcoumarin) in a spectrofluorometer (λex 380 nm; λem 460 nm). [file 1471-2091-12-61-S2.TIFF]

## Slide 1
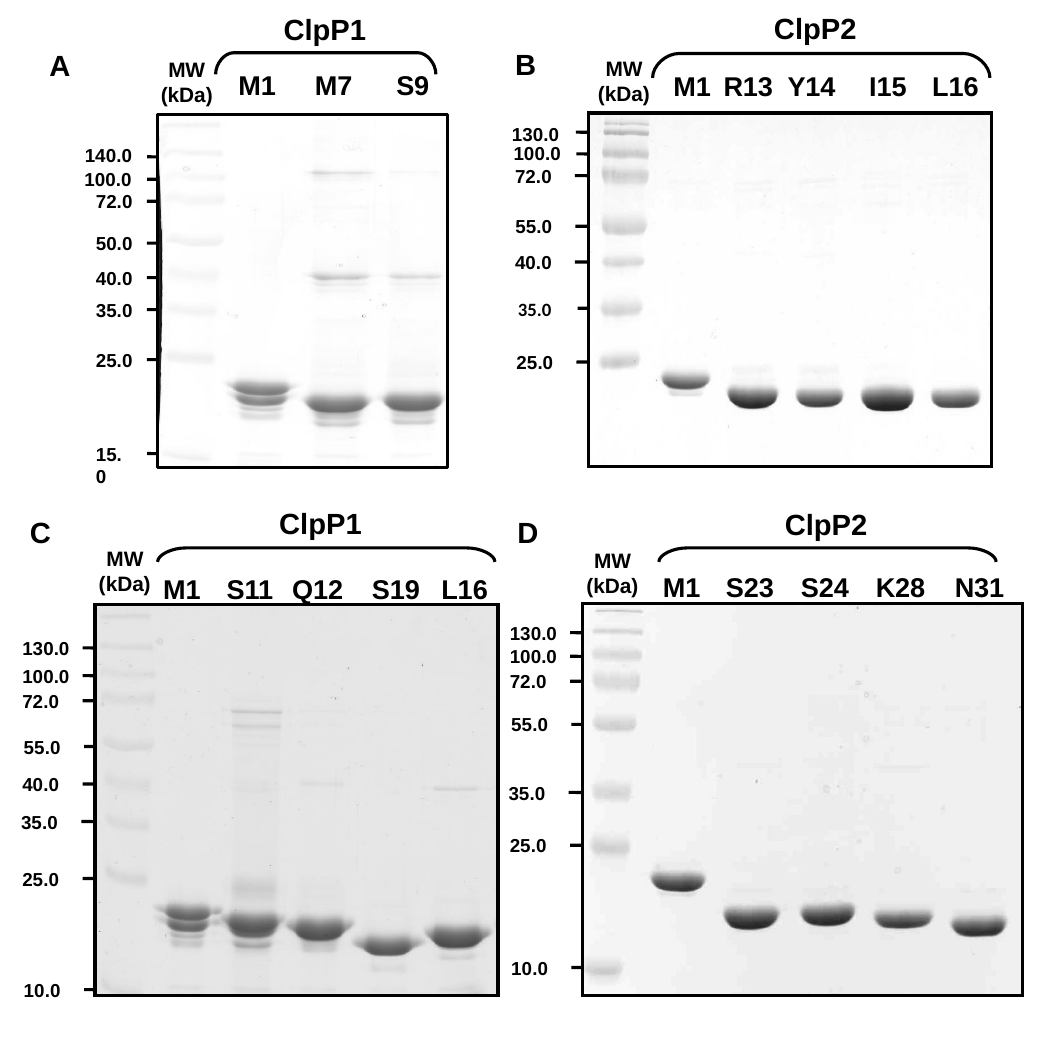

ClpP2
B
MW
(kDa)
M1
R13
Y14
I15
L16
130.0
100.0
72.0
55.0
40.0
35.0
25.0
ClpP1
A
MW
(kDa)
M1
M7
S9
140.0
100.0
72.0
50.0
40.0
35.0
25.0
15.0
ClpP1
C
MW
(kDa)
M1
S11
Q12
S19
L16
130.0
100.0
72.0
55.0
40.0
35.0
25.0
10.0
ClpP2
D
MW
(kDa)
M1
S23
S24
K28
N31
130.0
100.0
72.0
55.0
35.0
25.0
10.0

Supplement: Additional file 4 — Figure S4: Purified ClpP1 and ClpP2 variants. About 5 μg of the indicated purified proteins were loaded on a 12% SDS-PAGE stained with Coomassie blue. The molecular mass markers are indicated on the left. (A) Full length ClpP1 (M1) and the variants starting at the Met7 (M7) and Ser9 (S9). (B) Full length ClpP2 (M1) and the variants starting at Arg13 (R13), Tyr14 (Y14), Ile15 (I15), Leu16 (L16). (C) Full length ClpP1 (M1) and the variants starting at the Ser11 (S11), Gln12 (Q12), Leu16 (L16), and Ser19 (S19). (D) Full length ClpP2 (M1) and the variants starting at the Ser23 (S23), Ser24 (S24), Lys28 (K28), and Asn31 (N31). [file 1471-2091-12-61-S4.PPT]

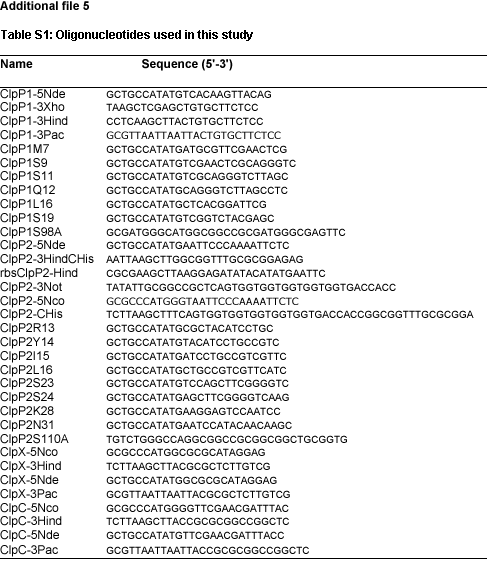

Supplement: Additional file 5 — Table S1: Oligonucleotides used in this study. [file 1471-2091-12-61-S5.TIFF]
