# Supplementary material for: Assembly and proteolytic processing of mycobacterial ClpP1 and ClpP2
Source: BMC Biochem. 2011 Dec 1;12:61. doi: 10.1186/1471-2091-12-61 (PMC3258218; doi:10.1186/1471-2091-12-61)
Supplement: Additional file 3 — Figure S3: Interaction of E. coli ClpP with ClpP1 or ClpP2. Soluble extracts were prepared from SG1146a cells producing untagged E. coli ClpP alone or together with ClpP1(His)6 or ClpP2(His)6 and loaded on a Ni2+ column. After extensive washing with buffer A (50 mM NaH2PO4 pH 8.0, 300 mM NaCl, 10 mM Imidazole, 10% glycerol), resin-bound proteins were eluted with buffer B (50 mM NaH2PO4 pH 8.0, 300 mM NaCl, 250 mM Imidazole, 10% glycerol). The presence of E. coli ClpP was analyzed by a 15% SDS-PAGE and detected by immunoblot using an anti ClpP antibody that exhibited cross-reaction with ClpP1 and ClpP2. (A) The indicated samples were loaded on a 15% SDS-PAGE stained with Coomassie blue. The molecular mass markers are indicated on the left. Lanes 1-3: 10 μg of the soluble extract of SG1146a cells producing untagged E. coli ClpP (lane 1), ClpP1(His)6 (lane 2), or untagged E. coli ClpP together with ClpP1(His)6 (lane 3) that were loaded on the Ni2+ column. Lanes 4-6: proteins eluted from Ni2+ column when SG1146a cells produced untagged E. coli ClpP (lane 4), ClpP1(His)6 (lane 5), or untagged E. coli ClpP together with ClpP1(His)6 (lane 6). The upper band in lane 6 is ClpP1(His)6 as determined by anti His tag immunoblot (data not shown) and the lower band is E. coli ClpP as determined by anti ClpP immunodetection in the panel (C). (B) The indicated samples were loaded on a 15% SDS-PAGE stained with Coomassie blue. The molecular mass markers are indicated on the left. Lanes 1-3: 10 μg of the soluble extract of SG1146a cells producing untagged E. coli ClpP (lane 1), ClpP2(His)6 (lane 2), or untagged E. coli ClpP together with ClpP2(His)6 (lane 3) that were loaded on the Ni2+ column. Lanes 4-6: proteins eluted from Ni2+ column when SG1146a cells produced untagged E. coli ClpP (lane 4), ClpP2(His)6 (lane 5), or untagged E. coli ClpP together with ClpP2(His)6 (lane 6). The upper band in lane 6 is ClpP2(His)6 as determined by anti His tag immunoblot (data not shown) and the lo [file 1471-2091-12-61-S3.PPT]

## Slide 1
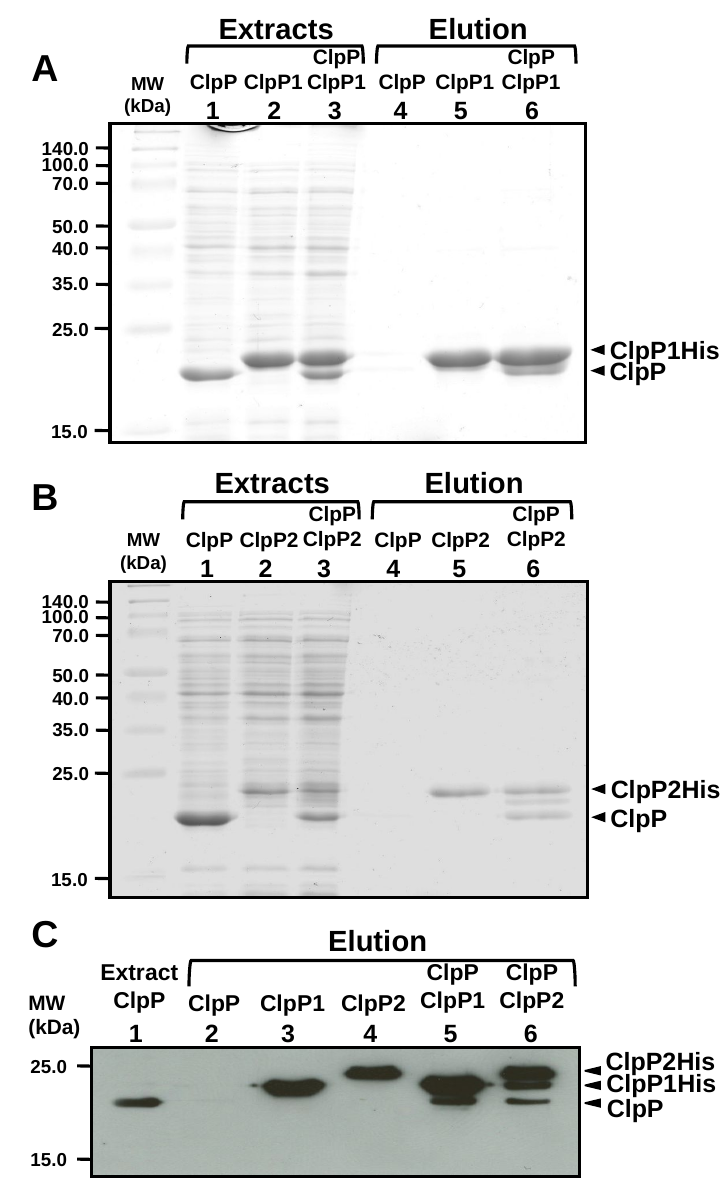

Extracts
Elution
ClpP
ClpP1
ClpP
ClpP1
ClpP
ClpP1
ClpP
ClpP1
MW
(kDa)
70.0
50.0
40.0
35.0
25.0
ClpP1His
ClpP
15.0
140.0
100.0
A
1
2
3
4
5
6
Extracts
Elution
ClpP
ClpP2
ClpP
ClpP2
ClpP
ClpP2
ClpP
ClpP2
MW
(kDa)
140.0
100.0
70.0
50.0
40.0
35.0
25.0
ClpP2His
ClpP
15.0
B
1
2
3
4
5
6
C
Elution
ClpP
ClpP1
ClpP
ClpP2
Extract
ClpP
ClpP
ClpP1
ClpP2
ClpP2His
25.0
ClpP1His
ClpP
15.0
MW
(kDa)
1
2
3
4
5
6
